# Association of polymorphism rs1053005 in *STAT3* with chronic hepatitis B virus infection in Han Chinese population

**DOI:** 10.1186/s12881-018-0569-x

**Published:** 2018-04-02

**Authors:** Man Li, Fang Li, Na Li, Jiao Sang, Xiude Fan, Huan Deng, Xiaoge Zhang, Qunying Han, Yi Lv, Zhengwen Liu

**Affiliations:** 1 Department of Infectious Diseases, First Affiliated Hospital of Xi’an Jiaotong University, No. 277 Yanta West Road, Xi’an, 710061 Shaanxi Province People’s Republic of China; 2 Department of Internal Medicine, Xi’an No. 3 Hospital, Xi’an, 710018 Shaanxi People’s Republic of China; 3 Department of Hepatobiliary Surgery, First Affiliated Hospital of Xi’an Jiaotong University, Xi’an, 710061 Shaanxi People’s Republic of China; 4 Institute of Advanced Surgical Technology and Engineering, Xi’an Jiaotong University, Xi’an, 710061 Shaanxi People’s Republic of China

**Keywords:** Hepatitis B virus, *STAT3*, rs1053004, rs1053005, Clinical disease

## Abstract

**Background:**

Signal transducer and activator of transcription 3 (STAT3) is involved in hepatitis B virus (HBV) infection and HBV-related hepatocellular carcinoma (HCC). The association between polymorphism rs1053005 and haplotypes formed by rs1053004 and rs1053005 in the 3′UTR of *STAT3* and chronic HBV infection has yet to be investigated.

**Methods:**

This study included 567 patients with chronic HBV infection (239 chronic hepatitis, 141 liver cirrhosis and 187 HCC), 98 HBV infection resolvers, and 169 healthy controls. *STAT3* rs1053004 and rs1053005 polymorphisms were genotyped by TaqMan SNP Genotyping Assays.

**Results:**

The rs1053004 genotype CC [*P* value by Bonferroni correction (*P*_*c*_) = 0.002] and allele C (*P*_*c*_ = 0.019) were more frequent in patients with chronic HBV infection than in healthy controls. The rs1053005 genotype GG was also more frequent in patients with chronic HBV infection than in healthy controls (*P*_*c*_ = 0.046). The rs1053004-rs1053005 haplotype T-G was less frequent in patients with chronic HBV infection than in healthy controls (*P*_c_ < 0.001). Haplotype C-A was more frequent in patients with liver cirrhosis than in patients with HCC (*P*_*c*_ = 0.042). The rs1053004 genotype TC, rs1053005 genotype AG and rs1053004-rs1053005 haplotype T-A were associated with higher HBV DNA levels.

**Conclusions:**

*STAT3* rs1053004 and rs1053005 polymorphisms and haplotypes formed by rs1053004 and rs1053005 are associated with chronic HBV infection and the haplotypes appear to be also associated with the development of liver disease. Studies in large sample sizes of patients and control populations are required to verify and extend these findings.

**Electronic supplementary material:**

The online version of this article (10.1186/s12881-018-0569-x) contains supplementary material, which is available to authorized users.

## Background

Hepatitis B virus (HBV) infection, which can result in acute and chronic hepatitis, liver cirrhosis and hepatocellular carcinoma (HCC), remains a worldwide public health issue [1, 2].

The intensity of immuno-inflammatory cells and cytokines is involved in the disease course and the development of various clinical consequences of HBV infection [3–5]. Signal transducer and activator of transcription 3 (STAT3) plays a key role in liver inflammation and cancer [6]. In HBV infection, dysregulated STAT3 signaling has been revealed to be involved in the ineffective immune response against HBV [7, 8] and the pathogenesis of liver diseases [7, 9, 10] through mediating the cytokine-mediated HBV enhancer function [9] and influencing the cytoprotective effect of hepatocyte growth factor and epidermal growth factor on CD95-mediated apoptosis and the action of cytotoxic T-cells [10]. STAT3 has also been shown to be involved in the enhanced Th17 response in acute-on-chronic liver failure (ACLF) associated with HBV infection [11] and the HBV reactivation in liver after radiotherapy [12].

It is, therefore, reasonable to hypothesize that polymorphisms in *STAT3* may have predisposing effects on the susceptibility to HBV infection and HBV-related liver diseases. Polymorphisms in the 3′ untranslated region (UTR) may be functionally relevant because this region usually contains regulatory elements that may post-transcriptionally regulate gene expression. There are 4 common single nucleotide polymorphisms (SNPs) (rs1053004, rs1053005, rs1053023 and rs3744483) with minor allele frequency (MAF) greater than 0.05 in the 3’ UTR of *STAT3* [13]. Of these polymorphisms, rs1053004 has been investigated in chronic HBV infection and HBV-related HCC in Chinese [14] and Thai populations [15], suggesting that this SNP might contribute to HCC susceptibility. However, the potential association of the remaining SNPs (rs1053005, rs1053023 and rs3744483) with chronic HBV infection remains to be investigated. Because these 3 SNPs are in perfect linkage disequilibrium [13], the present study investigated the association of *STAT3* rs1053005 polymorphism with chronic HBV infection in Han Chinese population. Haplotypes formed by rs1053004 and rs1053005 SNPs were also tested to provide complete information about the impact of 3’UTR SNPs of *STAT3* gene on HBV outcomes.

## Methods

### Study population

This study recruited 567 patients with chronic HBV infection, 98 HBV infection resolvers and 169 healthy controls. The patients with chronic HBV infection were recruited from the First Affiliated Hospital of Xi’an Jiaotong University, a large tertiary hospital in Xi’an, Shaanxi province, northwest China. The clinical diagnoses of the 567 patients with chronic HBV infection included 239 chronic hepatitis, 141 liver cirrhosis and 187 HCC according to the history of infection, liver biochemistry, HBV markers and liver imaging as described previously [16]. Other hepatic viral infection (hepatitis A virus, hepatitis C virus, hepatitis D virus, or hepatitis E virus), autoimmune hepatitis and drug-induced hepatitis or alcoholic hepatitis, concurrent infection of human immunodeficiency virus-1 were all excluded in the patients. The 98 HBV infection resolvers and the 169 healthy controls were recruited from the persons who received physical examinations at the First Affiliated Hospital of Xi’an Jiaotong University. The HBV infection resolvers were those who spontaneously recovered from HBV infection and had seropositivity of anti-HBs and anti-HBc, seronegativity of HBsAg and HBeAg, undetectable serum HBV DNA, and normal liver biochemistry. The healthy controls were free of HBV infection and had no history of liver disease. All the participants are ethnically Han Chinese and geographically permanent residents of Shaanxi Province, northwest China.

### Genotyping of rs1053004 and rs1053005 polymorphisms

Genomic DNA was directly extracted from EDTA-treated peripheral blood using TIANamp Genomic DNA Kit [Tiangen Biotech (Beijing) Co., Ltd., Beijing, China] according to manufacturer’s instruction. *STAT3* rs1053004 and rs1053005 polymorphisms were genotyped by TaqMan MGB high throughput RT-PCR method on the ABI 7500 fast real-time PCR platform (Applied Biosystems, ABI Technologies, USA). The results were analyzed on 7500 Fast System V1.4.0 SDS software. For quality control, the genotyping was repeated once with concordance rate of 100% in the samples included in the study.

### Statistical analysis

Statistical tests were conducted with SPSS 16.0 (SPSS, Inc., Chicago, IL). *P* value < 0.05 was regarded as statistically significant. Multiple testing correction was performed by the Bonferroni method. The chi-square test or student’s t-test was used to compare parameters between groups. The frequency of genotypes and alleles was determined by direct gene counting method. The chi-square test or Fisher’s exact test was used to estimate the Hardy–Weinberg equilibrium (HWE) and to compare the genotype and allele frequencies between the groups. Odds ratios (OR) with 95% confidence interval (CI) were calculated by logistic regression analyses adjusted for age and sex. Haplotype analysis was performed using SHEsis platform [17, 18].

## Results

### Characteristics and hardy-Weinberg equilibrium of the genotypes of rs1053004 and rs1053005 polymorphism in the study subjects

The demographics of the study participants are shown in Table 1. The gender and age between patients with chronic HBV infection, HBV infection resolvers and healthy controls were comparable (*P =* 0.159 and *P =* 0.084, respectively. Table 1). The genotype distribution of *STAT3* rs1053004 and rs1053005 polymorphisms in patients with chronic HBV infection (*P* = 0.204 and *P* = 0.687, respectively), HBV infection resolvers (*P* = 0.509 and *P* = 0.487, respectively) and healthy controls (*P* = 0.059 and *P* = 0.052, respectively) were accorded with Hardy-Weinberg equilibrium (Table 1).

**Table 1:** Demographics and Hardy-Weinberg equilibrium (HWE) of the genotypes of *STAT3* rs1053004 and rs1053005 in patients with chronic hepatitis B virus (HBV) infection, HBV infection resolvers and healthy controls

	Patients with chronic HBV infection (*n* = 567)	HBV infection resolvers (*n* = 98)	Healthy controls (*n* = 169)	*P*
Gender(Male/Female)	384/183	64/34	101/68	0.159
Age (year)	41.10 ± 12.96	40.11 ± 13.17	43.33 ± 13.27	0.084
rs1053004 HWE				
*P*	0.204	0.509	0.059	
rs1053005 HWE				
*P*	0.687	0.487	0.052	

### Genotype, allele and haplotype frequencies of rs1053004 and rs1053005 polymorphisms in patients with chronic HBV infection, HBV infection resolvers and healthy controls

The genotype and allele frequencies of the *STAT3* rs1053004 and rs1053005 polymorphisms in the study populations are presented in Table 2. Using genotype TT as reference, the frequency of rs1053004 genotype TC between patients, infection resolvers and controls had no significant differences. However, the frequency of rs1053004 genotype CC in patients was significantly higher than in healthy controls [11.6% vs. 2.9%, *P* = 0.001, *P* value by Bonferroni correction (*P*_*c*_) = 0.002, OR 4.367, 95%CI 1.705–11.185] although its frequency between HBV infection resolvers and patients had no significant difference (Table 2). The frequency of rs1053004 allele C was not significantly different between patients and infection resolvers, but this allele was significantly more frequent in patients than in healthy controls (32.3% vs. 25.4%, *P* = 0.019, *P*_*c*_ = 0.019, OR 1.402, 95%CI, 1.065–1.845, Table 2).

**Table 2:** Genotype, allele and haplotype frequencies of *STAT3* rs1053004 and rs1053005 polymorphisms in patients with chronic hepatitis B virus (HBV) infection, HBV infection resolvers and healthy controls

*STAT3* polymorphism	Patients (n = 567)	Resolvers (n = 98)	Controls (n = 169)	Patients vs. Resolvers		Patients vs. Controls	
				*P*	OR (95%CI)	*P*	OR (95%CI)
rs1053004							
Genotype							
TT	266 (46.9)	42 (42.9)	88 (52.1)	Ref.		Ref.	
TC	235 (41.4)	42 (42.9)	76 (45.0)	0.599	0.883 (0.556–1.403)	0.900	0.978 (0.686–1.392)
CC	66 (11.6)	14 (14.2)	5 (2.9)	0.381	0.744 (0.384–1.443)	0.001 ^a^	4.367 (1.705–11.185)
Allele							
T	767 (67.6)	126 (64.3)	252 (74.6)	Ref.		Ref.	
C	367 (32.3)	70 (35.7)	86 (25.4)	0.356	0.861 (0.627–1.183)	0.019 ^b^	1.402 (1.065–1.845)
rs1053005							
Genotype							
AA	271 (47.8)	44 (44.9)	84 (49.7)	Ref.		Ref.	
AG	239 (42.2)	41 (41.8)	78 (46.2)	0.814	1.057 (0.667–1.673)	0.775	0.950 (0.667–1.353)
GG	57 (10.0)	13 (13.3)	7 (4.1)	0.327	0.712 (0.360–1.407)	0.023^c^	2.524 (1.109–5.744)
Allele							
A	781(68.9)	129 (65.8)	246 (72.8)	Ref.			
G	353 (31.1)	67 (34.2)	92 (27.2)	0.208	0.870 (0.631–1.199)	0.170	1.209 (0.922–1.584)
Haplotype (rs1053004- rs1053005)							
T-A	767 (67.7)	126 (64.8)	246 (72.8)	Ref.		Ref.	
C-G	353 (31.1)	67 (34.2)	86 (25.4)	0.379	0.866 (0.627–1.194)	0.059	1.316 (0.999–1.735)
T-G	0 (0)	0 (0)	6 (1.8)	1.000	1.000 (1.000–1.000)	< 0.001^d^	0.243 (0.218–0.271)
C-A	14 (1.2)	3 (1.0)	0 (0)	0.950	1.304 (0.370–4.604)	0.034^e^	1.321 (1.275–1.368)

Data are presented as n (%). *OR* odds ratio, *95%CI* 95% confidence interval, *Ref.* reference. ^a^ *P* value by Bonferroni correction = 0.002; ^b^*P* value by Bonferroni correction = 0.019; ^c^*P* value by Bonferroni correction = 0.046; ^d^*P* value by Bonferroni correction< 0.001; ^e^*P* value by Bonferroni correction = 0.102

For rs1053005 polymorphism, patients with chronic HBV infection had higher frequency of genotype GG than healthy controls (10.0% vs. 4.1%, *P* = 0.023, *P*_*c*_ = 0.046, OR 2.524, 95%CI 1.109–5.744, Table 2). The allele frequency of rs1053005 polymorphism between patients, resolvers and healthy controls had no significant differences (Table 2).

Haplotype analysis showed that, using rs1053004-rs1053005 haplotype T-A as reference, the frequency of haplotype C-G had no significant difference between patients and resolvers and between patients and healthy controls. Haplotype T-G was less frequent in patients than in healthy controls (0% vs. 1.8%, *P* < 0.001, *P*_*c*_ < 0.001, OR 0.243, 95%CI 0.218–0.271). Haplotype C-A was more frequent in patients than in healthy controls but the difference was not significant by Bonferroni correction (1.2% vs. 0%, *P* = 0.034, *P*_*c*_ = 0.102, OR 1.321, 95%CI 1.275–1.368, Table 2).

### Genotype, allele and haplotype frequencies of rs1053004 and rs1053005 polymorphisms in HBV infection resolvers and patients with chronic hepatitis

The genotype, allele and haplotype frequencies of the *STAT3* rs1053004 and rs1053005 polymorphisms in HBV infection resolvers and patients with chronic hepatitis were compared. No significant differences in the genotype, allele and haplotype frequencies of rs1053004 and rs1053005 polymorphisms were observed between HBV infection resolvers and chronic hepatitis patients (Table 3).

**Table 3:** Genotype, allele and haplotype (rs1053004-rs1053005) frequencies of *STAT3* rs1053004 and rs1053005 polymorphisms in hepatitis B virus infection resolvers and patients with chronic hepatitis

*STAT3* polymorphism	Resolvers (n = 98)	CH (*n* = 239)	*P*	OR (95%CI)
rs1053004				
Genotype				
TT	42 (42.9)	108 (45.2)	Ref.	
TC	42 (42.9)	103 (43.1)	0.854	0.954 (0.575–1.581)
CC	14 (14.2)	28 (11.7)	0.501	1.286 (0.617–2.678)
Allele				
T	126 (64.3)	319 (66.7)	Ref.	
C	70 (35.7)	159 (33.3)	0.542	1.115 (0.786–1.580)
rs1053005				
Genotype				
AA	44 (44.9)	110 (46.0)	Ref.	
AG	41 (41.8)	104 (43.5)	0.814	0.946 (0.598–1.499)
GG	13 (13.3)	25 (10.5)	0.327	1.405 (0.711–2.777)
Allele				
A	129 (65.8)	324 (67.8)	Ref.	
G	67 (34.2)	154 (32.2)	0.396	1.149 (0.834–1.584)
Haplotype				
T-A	126 (64.8)	319 (66.7)	Ref.	
C-G	67 (34.2)	154 (32.2)	0.592	1.101 (0.774–1.568)
T-G	0 (0)	0 (0)	1.000	1.000 (1.000–1.000)
C-A	3 (1.0)	5 (1.2)	0.568	0.658 (0.155–2.796)

Data are presented as n (%). *CH* chronic hepatitis, *OR* odds ratio, *95%CI* 95% confidence interval, *Ref.* reference

### Genotype, allele and haplotype frequencies of rs1053004 and rs1053005 polymorphisms in patients with chronic hepatitis, liver cirrhosis and HCC

The genotype and allele frequencies of the *STAT3* rs1053004 and rs1053005 polymorphisms in HBV infected patients with chronic hepatitis, liver cirrhosis and HCC are shown in Table 4. There were no significant differences in the genotype and allele frequencies of both rs1053004 and rs1053005 polymorphisms between HBV infected patients with chronic hepatitis, liver cirrhosis and HCC (Table 4).

**Table 4:** Genotype, allele and haplotype frequencies of *STAT3* rs1053004 and rs1053005 polymorphisms in patients with chronic hepatitis, liver cirrhosis and hepatocellular carcinoma

*STAT3* polymorphism	CH (n = 239)	LC (*n* = 141)	HCC (*n* = 187)	CH vs. LC		CH vs. HCC		LC vs. HCC	
				*P*	OR (95%CI)	*P*	OR (95%CI)	*P*	OR (95%CI)
rs1053004									
Genotype									
TT	108 (45.2)	76 (53.9)	82 (43.9)	Ref.		Ref.		Ref.	
TC	103 (43.1)	50 (35.4)	82 (43.9)	0.103	0.690 (0.441–1.079)	0.820	1.049 (0.697–1.577)	0.103	1.520 (0.950–2.432)
CC	28 (11.7)	15 (10.6)	23 (12.2)	0.439	1.314 (0.657–2.625)	0.804	0.924 (0.496–1.721)	0.338	0.704 (0.342–1.448)
Allele									
T	319 (66.7)	202 (71.6)	246 (65.8)	Ref.		Ref.		Ref.	
C	159 (33.3)	80 (28.4)	128 (34.2)	0.160	1.259 (0.913–1.735)	0.768	1.044 (0.784–1.390)	0.111	0.761 (0.544–1.065)
rs1053005									
Genotype									
AA	110 (46.0)	78 (55.3)	83 (44.4)	Ref.		Ref.		Ref.	
AG	104 (43.5)	51 (36.2)	84 (44.9)	0.102	1.446 (0.928–2.252)	0.742	0.934 (0.623–1.400)	0.065	0.646 (0.406–1.029)
GG	25 (10.5)	12 (8.5)	20 (10.7)	0.304	1.477 (0.700–3.118)	0.861	0.943 (0.491–1.813)	0.257	0.638 (0.293–1.392)
Allele									
A	324 (67.8)	207 (73.4)	250 (66.8)	Ref.		Ref.		Ref.	
G	154 (32.2)	75 (26.6)	124 (33.2)	0.103	1.312 (0.946–1.818)	0.772	0.958 (0.718–1.279)	0.070	1.369 (0.974–1.925)
Haplotype (rs1053004-rs1053005)									
T-A	319 (66.7)	202 (71.6)	246 (65.8)	Ref.		Ref.		Ref.	
C-G	154 (32.2)	75 (26.6)	124 (33.2)	0.116	1.300 (0.937–1.804)	0.770	1.044 (0.782–1.394)	0.079	1.358 (0.965–1.911)
T-G	0 (0)	0 (0)	4 (1.8)	1.000	1.000 (1.000–1.000)	0.023^a^	0.435 (0.396–0.478)	0.025^b^	0.549 (0.505–0.597)
C-A	5 (1.2)	5 (1.0)	0 (0)	0.471	1.579 (0.452–5.523)	0.050^c^	1.771 (1.647–1.904)	0.014^d^	2.218 (2.002–2.456)

Data are presented as n (%). *CH* chronic hepatitis, *LC* liver cirrhosis, *HCC* hepatocellular carcinoma, *OR* odds ratio, *95%CI* 95% confidence interval, *Ref.* reference. ^a^ *P* value by Bonferroni correction = 0.069; ^b^*P* value by Bonferroni correction = 0.075; ^c^*P* value by Bonferroni correction = 0.150; ^d^*P* value by Bonferroni correction = 0.042

Haplotype analysis showed that, using rs1053004-rs1053005 haplotype T-A as reference, haplotype C-G frequency had no significant difference between HBV infected patients with chronic hepatitis, liver cirrhosis and HCC. However, haplotype T-G was less frequent in patients with chronic hepatitis and patients with liver cirrhosis than in patients with HCC (0% vs. 1.8%, *P* = 0.023, OR 0.435, 95% CI 0.396–0.478 and 0% vs. 1.8%, *P* = 0.025, OR 0.549, 95% CI 0.505–0.597, respectively) but these differences were not significant after Bonferroni correction (*P*_*c*_ = 0.069 and *P*_*c*_ = 0.075, respectively, Table 4). Haplotype C-A was more frequent in patients with liver cirrhosis than in patients with HCC (1.0% vs. 0%, *P* = 0.014, *P*_*c*_ = 0.042, OR 2.218, 95% CI, 2.002–2.456, respectively, Table 4).

### Associations between *STAT3* rs1053004 and rs1053005 polymorphisms and HBV DNA levels

**Fig. 1:**
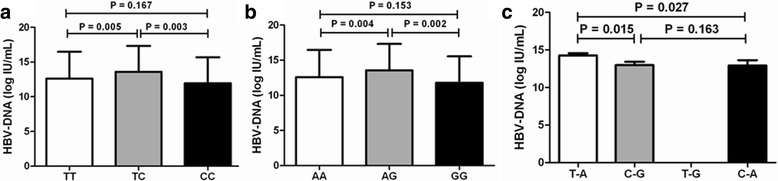
Associations of rs1053004 (**a**) and rs1053005 (**b**) genotypes and rs1053004-rs1053005 haplotypes (**c**) with HBV DNA levels in patients with chronic HBV infection

Correlation analysis between *STAT3* rs1053004 and rs1053005 polymorphisms and HBV DNA levels showed that rs1053004 genotype TC was associated with higher HBV DNA levels compared with genotypes TT and CC (*P* = 0.005 and *P* = 0.003, respectively, Fig. 1a, Additional file 1: Table S1). The rs1053005 genotype AG was also associated with higher HBV DNA levels compared to genotypes AA and GG (*P* = 0.004 and *P* = 0.002, respectively, Fig. 1b, Additional file 1: Table S1). Patients carrying haplotype T-A had higher HBV DNA levels than those carrying haplotype C-G and those carrying haplotype C-A (*P* = 0.015 and *P* = 0.027, respectively) and HBV DNA levels between patients carrying haplotype C-G and those carrying haplotype C-A were not significantly different (Fig. 1c, Additional file 1: Table S1).

## Discussion

There are 4 common SNPs (rs1053004, rs1053005, rs1053023 and rs3744483) with MAF greater than 0.05 in the 3′UTR of *STAT3* [13]. The SNP rs1053004 has been investigated in HBV infection and is suggested to be associated with HBV-related HCC in Chinese and Thai populations [14, 15]. The role of rs1053005, rs1053023 and rs3744483 in chronic HBV infection remains uninvestigated. Because these 3 SNPs are in perfect linkage disequilibrium [13], this study examined the possible association of rs1053005 polymorphism and the haplotypes formed by rs1053004 and rs1053005 polymorphisms with chronic HBV infection in Han Chinese population. The results showed that patients with chronic HBV infection had significantly higher frequency of rs1053004 genotype CC and allele C than healthy controls. Patients with chronic HBV infection also had higher frequency of rs1053005 genotype GG than healthy controls. Patients with chronic HBV infection had less frequent rs1053004-rs1053005 haplotype T-G than healthy controls. In regard with clinical disease, haplotype C-A was more frequent in patients with liver cirrhosis than in patients with HCC. Namely, HCC patients had lower frequency of haplotype C-A than patients with liver cirrhosis. The rs1053004 genotype TC, rs1053005 genotype AG and haplotype T-A were associated with higher HBV DNA levels.

*STAT3* rs1053004 has been suggested to be involved in chronic HBV infection in Chinese [14] and Thai populations [15]. *STAT3* rs1053005 has been shown to be associated with ankylosing spondylitis and autoimmune thyroid disease in Han Chinese population [19, 20], to affect the risk of obesity and hypertriglyceridemia in Chinese Han population and French [21, 22], and to contribute to anti-tuberculosis drug-induced hepatitis (ATDH) susceptibility in Chinese Han population [23]. It is implied that this SNP is immunologically, metabolically and hepatologically relevant. Insufficient immunological response is a major characteristic of chronic HBV infection and HBV-related liver disease [3–5]. The present study showed that patients with chronic HBV infection had significantly higher frequency of rs1053004 genotype CC and allele C, higher frequency of rs1053005 genotype GG, and lower frequency of haplotype T-G than healthy controls. These results indicated that both rs1053004 and rs1053005 polymorphisms may play a role in chronic HBV infection.

Previous studies showed that *STAT3* SNPs rs4796793, rs2293152, and rs1053004 were interactively involved in the hepatocarcinogenesis in the host with HBV mutations in Han Chinese [14]. The *STAT3* SNP rs1053004 was also shown to be contributable to HCC susceptibility in the Thai population [15]. The SNP rs111904020 (U/G) in *STAT3* 3’UTR was shown to act as a promotion factor in HCC development in Chinese population [24]. In the present study, although genotype and allele analyses did not show relevance between rs1053004 and rs1053005 polymorphisms and HBV infected patients with different liver diseases including HCC. Haplotype analysis showed that HCC patients had lower frequency of haplotype C-A than patients with liver cirrhosis. These results suggested that rs1053004 and rs1053005 polymorphisms are possibly interactively involved in the hepatocarcinogenesis of chronic HBV infection. Notably, regarding the susceptibility to chronic HBV infection in relation to healthy controls and the susceptibility to HCC in relation to other liver disease such as liver cirrhosis in HBV infection, the susceptible haplotypes were differentially distributed because patients with chronic HBV infection had less frequent haplotype T-G than healthy controls while HCC patients had lower frequency of haplotype C-A than patients with liver cirrhosis. Furthermore, this study indicated that genotypes, namely rs1053004 genotype TC and rs1053005 genotype AG, associated with higher levels of HBV DNA, are both heterozygous. Haplotype T-A was also associated with higher HBV DNA levels. It is suggested that the associations of *STAT3* polymorphisms with the susceptibility of chronic HBV infection and the development of liver disease including HCC are complicated.

There are other SNPs in *STAT3* which have been revealed to be associated with human disease. For example, *STAT3* rs3816769 and rs744166 polymorphisms were shown to play a significant role in susceptibility to autoimmune thyroid disease Hashimoto’s thyroiditis and Graves’ disease in the Polish population [25]. Therefore, more SNPs in *STAT3* need to be genotyped in chronic HBV infection and HBV-related liver disease including HCC to clarify the detailed profile of *STAT3* polymorphisms in HBV infection in future studies.

The present study was conducted in small sample sizes of patients and controls, genotyped and analyzed only two polymorphisms in the 3’ UTR of *STAT3*, and did not include replicative patient and control populations to confirm the findings. Therefore, the results appear to be very preliminary. Further studies in large patient and control populations with more polymorphisms genotyped are definitely needed to elucidate the possible role of *STAT3* polymorphisms in chronic HBV infection and HBV-related liver diseases.

## Conclusions

This study indicates that *STAT3* rs1053004 and rs1053005 polymorphisms and haplotypes formed by rs1053004 and rs1053005 are associated with chronic HBV infection and the haplotypes appear to be also associated with the development of liver disease. Studies in large sample sizes of patient and control populations are required to confirm and extend these findings. Studies are also needed to elucidate the underlying mechanisms related to the potential association between *STAT3* polymorphisms and chronic HBV infection and HBV-related liver diseases.

## Additional file

Additional file 1: **Table S1.** Associations of *STAT3* rs1053004 and rs1053005 polymorphisms and rs1053004-rs1053005 haplotypes with HBV DNA levels. (DOC 101 kb)

## Abbreviations

ACLF: Acute-on-chronic liver failure
ATDH: Anti-tuberculosis drug-induced hepatitis
HBV: Hepatitis B virus
HCC: Hepatocellular carcinoma
HWE: Hardy–Weinberg equilibrium
MAF: Minor allele frequency
SNP: Single nucleotide polymorphism
STAT3: Signal transducer and activator of transcription 3
UTR: Untranslated region
