# Supplementary material for: Association of polymorphism rs1053005 in STAT3 with chronic hepatitis B virus infection in Han Chinese population
Source: BMC Med Genet. 2018 Apr 2;19:52. doi: 10.1186/s12881-018-0569-x (PMC5879595; doi:10.1186/s12881-018-0569-x)
Supplement: Supplementary file 1 — Table S1. Associations of STAT3 rs1053004 and rs1053005 polymorphisms and rs1053004-rs1053005 haplotypes with HBV DNA levels. (DOC 101 kb) [file 12881_2018_569_MOESM1_ESM.doc]

Table S1. Associations of *STAT3* rs1053004 and rs1053005 polymorphisms and rs1053004-rs1053005 haplotypes with HBV DNA levels.

| *STAT3* polymorphism | HBV DNA (log IU/mL) |
| --- | --- |
| rs1053004 |  |
| TT | 11.63±3.86 |
| TC | 14.59±2.66 |
| CC | 10.57±1.22 |
| rs1053005 |  |
| AA | 12.09±2.53 |
| AG | 14.33±3.13 |
| GG | 11.06±1.96 |
| Haplotype (rs1053004-rs1053005) |  |
| T-A | 14.97±0.32 |
| C-G | 12.06±1.03 |
| C-A | 12.27±0.85 |
